# Molecular Basis of Rare Diseases Associated to the Maturation of Mitochondrial [4Fe-4S]-Containing Proteins

**DOI:** 10.3390/biom12071009

**Published:** 2022-07-21

**Authors:** Francesca Camponeschi, Simone Ciofi-Baffoni, Vito Calderone, Lucia Banci

**Affiliations:** 1 Magnetic Resonance Center CERM, University of Florence, 50019 Sesto Fiorentino, Italy; camponeschi@cerm.unifi.it (F.C.); banci@cerm.unifi.it (L.B.); 2 Consorzio Interuniversitario Risonanze Magnetiche di Metalloproteine (CIRMMP), 50019 Sesto Fiorentino, Italy; 3 Department of Chemistry, University of Florence, 50019 Sesto Fiorentino, Italy

**Keywords:** iron–sulfur cluster, mitochondrial proteins, multiple mitochondrial dysfunction syndromes, rare diseases

## Abstract

The importance of mitochondria in mammalian cells is widely known. Several biochemical reactions and pathways take place within mitochondria: among them, there are those involving the biogenesis of the iron–sulfur (Fe-S) clusters. The latter are evolutionarily conserved, ubiquitous inorganic cofactors, performing a variety of functions, such as electron transport, enzymatic catalysis, DNA maintenance, and gene expression regulation. The synthesis and distribution of Fe-S clusters are strictly controlled cellular processes that involve several mitochondrial proteins that specifically interact each other to form a complex machinery (Iron Sulfur Cluster assembly machinery, ISC machinery hereafter). This machinery ensures the correct assembly of both [2Fe-2S] and [4Fe-4S] clusters and their insertion in the mitochondrial target proteins. The present review provides a structural and molecular overview of the rare diseases associated with the genes encoding for the accessory proteins of the ISC machinery (i.e., GLRX5, ISCA1, ISCA2, IBA57, FDX2, BOLA3, IND1 and NFU1) involved in the assembly and insertion of [4Fe-4S] clusters in mitochondrial proteins. The disease-related missense mutations were mapped on the 3D structures of these accessory proteins or of their protein complexes, and the possible impact that these mutations have on their specific activity/function in the frame of the mitochondrial [4Fe-4S] protein biogenesis is described.

## 1. Introduction

Mitochondria play pivotal roles in mammalian cells. A number of processes occur within mitochondria, including ATP production, metabolic pathways, such as citric acid and urea cycles, biosynthesis of amino acids, lipids and of essential protein cofactors such as heme, biotin, lipoic acid, molybdenum cofactor, and iron–sulfur (Fe-S) clusters [1,2]. The latter are evolutionarily conserved, ubiquitous inorganic cofactors, performing a multiplicity of functions, such as electron transport, enzymatic catalysis, DNA maintenance, and gene expression regulation [3,4,5]. In mammalian cells, the synthesis and distribution of Fe-S clusters is a tightly controlled process, performed in mitochondria by several proteins that specifically interact each other to form a complex machinery, the so-called Iron Sulfur Cluster assembly machinery (ISC machinery hereafter) [6,7,8,9]. The latter ensures the correct assembly and insertion of Fe-S clusters in mitochondrial target proteins. 

**Figure 1:**
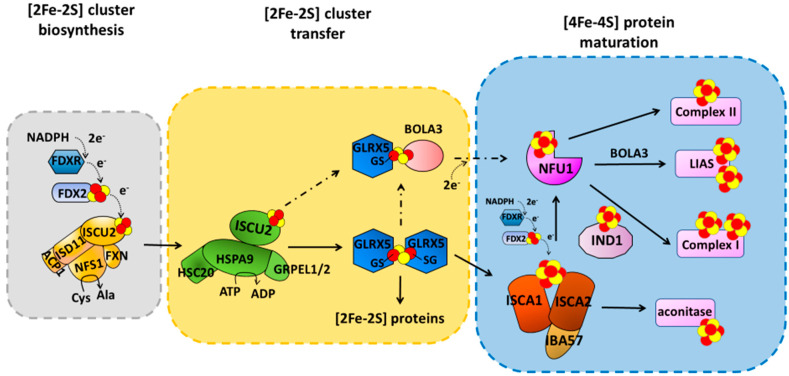
The three steps of the mitochondrial iron–sulfur cluster assembly machinery required to mature mitochondrial [4Fe-4S] target proteins. In the first step, a [2Fe-2S] cluster is assembled de novo on the scaffold protein ISCU2. The biosynthesis involves six additional ISC proteins including the cysteine desulfurase complex NFS1-ISD11-ACP1 as a sulfur donor, frataxin (FXN), and the electron (e^−^) transfer chain from NADPH via ferredoxin reductase (FDXR) to ferredoxin FDX2. Only one half of the symmetric core ISC complex is depicted. In the second step, a dedicated chaperone system (HSPA9, HSC20, and GRPLE1/2) facilitates the transfer of the [2Fe-2S] cluster from the ISCU2 scaffold to the monothiol glutaredoxin GLRX5, which binds the cluster in glutathione (GS)-dependent fashion. A secondary route (indicated by dashed arrows) involves BOLA3, which forms an apo complex with GLRX5 able to receive a [2Fe-2S] cluster from ISCU2 to form the homodimeric [2Fe-2S] GLRX5 complex; or, alternatively, the latter complex can be formed via the interaction of BOLA3 with the homodimeric [2Fe-2S] GLRX5 complex. The third step involves [4Fe-4S] cluster synthesis and apoprotein insertion. GLRX5 delivers its [2Fe-2S] cluster to three late-acting ISC proteins (ISCA1, ISCA2, and IBA57) for [4Fe-4S] cluster biosynthesis, which additionally requires the ferredoxin FDX2 electron transfer chain. Subsequently, the newly formed [4Fe-4S] cluster is delivered to recipient apoproteins by dedicated Fe-S-targeting proteins (e.g., NFU1, IND1 directly binding the [4Fe-4S] cluster) via parallel pathways. The major role of BOLA3 protein is in lipoyl synthase (LIAS) maturation. BOLA3 might be involved in such function via an alternative pathway, which consists on the [2Fe-2S] cluster donation from [2Fe-2S] GLRX5-BOLA3 complex to apo NFU1 to assemble a [4Fe-4S] cluster on NFU1 thanks to the delivery of two electrons, whose physiological source needs, however, to be identified.

The ISC machinery can be dissected into three main steps (Figure 1), the first of which is the de novo synthesis of a [2Fe-2S]^2+^ cluster on a multimeric protein complex, formed by the scaffold protein ISCU2, the cysteine desulfurase complex NFS1/ISD11/ACP1, and frataxin (FXN) [10,11,12,13]. The cluster is assembled from ferrous ions and inorganic sulfur. While the latter is provided by the cysteine desulfurase NFS1/ISD11/ACP1 complex that converts Cys into Ala [14,15], the entry mechanism of Fe^2+^ into the multimeric complex is still not clearly defined. Electrons are also required for the formation of the [2Fe-2S]^2+^ cluster and are provided by the mitochondrial ferredoxin (FDX2)/ferredoxin reductase (FDXR) system (Figure 1) [16,17,18]. The newly synthesized cluster is then released and transferred downstream of the ISCU2/NFS1/ISD11/ACP1 complex with the help of adaptors, such as HSPA9 and HSC20 [19], to the monothiol glutaredoxin GLRX5, for the following insertion into target [2Fe-2S]-binding proteins (Figure 1). An in vitro study recently showed that [2Fe-2S] ISCU2 can also transfer the cluster to apo GLRX5-BOLA3 complex [20] to form a heterodimeric [2Fe-2S] GLRX5-BOLA3 complex, which might have a secondary role in the following step of the machinery to assemble [4Fe-4S] clusters. Indeed, the main route of the ISC machinery involves the GLRX5-bound [2Fe-2S]^2+^ cluster that is used as a starting point to form a [4Fe-4S]^2+^ cluster. Specifically, the [2Fe-2S]^2+^ cluster bound to homodimeric GLRX5 is transferred to an accessory protein system able to assemble a [4Fe-4S]^2+^ cluster [21,22] and to insert it into recipient apoproteins (Figure 1). This accessory protein system forms the last step of the ISC machinery, involving several proteins (ISCA1, ISCA2, IBA57, FDX2, IND1, BOLA3, and NFU1) that work in parallel pathways, each maturing specific final target(s) (Figure 1) [6]. 

The correct functioning of the ISC machinery is of vital importance for the correct activity of mitochondria. Indeed, mutations in the genes encoding for the components of the ISC machinery cause rare but severe diseases in humans [23,24]. These include Friedreich’s ataxia (FRDA), a neurodegenerative disease caused by mutations in frataxin (FXN), myopathies caused by mutations in ISCU2 and FDX2, a rare form of sideroblastic anemia caused by mutation in the GLRX5 gene, a form of encephalomyopathy caused by dysfunction of the respiratory chain complex I as a consequence of mutation in the gene encoding for IND1, and multiple mitochondrial dysfunction syndromes (MMDSs) caused by mutations in the genes encoding for the proteins acting in the last step of the ISC machinery (see later).

Here, we present a report describing, from a structural and molecular perspective, the rare diseases associated with the genes encoding for the proteins involved in the assembly of [4Fe-4S] clusters in mitochondria, i.e., GLRX5, ISCA1, ISCA2, IBA57, FDX2, BOLA3, IND1 and NFU1. Specifically, we mapped the disease-related missense mutations on the 3D structures of these proteins or of their protein complexes, and discussed the possible impact that these mutations have on their specific activity/function in the frame of the mitochondrial [4Fe-4S] protein biogenesis. 

## 2. [4Fe-4S] Cluster Assembly in Mitochondria

The mechanism responsible for the maturation of mitochondrial [4Fe-4S] proteins is not yet fully defined. Indeed, both transient protein–protein interactions and stable protein complexes involved in such processes are still only partially characterized at the molecular level, as well as the picture of the operative protein–protein interaction network is still argument of debate in the literature, where two main models have been proposed. One model proposed that NFU1 operates with ISCU2 and ISCA1 proteins to assemble a [4Fe-4S]^2+^ cluster [25]. Specifically, the latter proteins provide a [2Fe-2S]^2+^ cluster each to NFU1 forming a ISCU2-ISCA1-NFU1 complex; this ternary complex receives two electrons from FDX2 to assemble a [4Fe-4S]^2+^ cluster on dimeric NFU1 [25]. The [4Fe-4S]^2+^ cluster assembled on NFU1 was proposed to be then transferred to apo recipient proteins [25]. However, at the moment, the most accredited model, based on experimental data collected by different research groups, supports a different protein–protein interaction network, described hereafter. This model has been here considered in the description of the effects that pathogenic mutations have on the maturation of mitochondrial [4Fe-4S] proteins.

ISCA1 and ISCA2 preferentially form a stable hetero-dimeric complex responsible for assembling a [4Fe-4S] cluster [21,26]. The latter is assembled through a reductive coupling of two [2Fe-2S] clusters that are donated to the apo ISCA1-ISCA2 hetero-complex by homo-dimeric [2Fe-2S]^2+^ GLRX5 (Figure 1) [21,22,27]. The latter bridges a [2Fe-2S]^2+^ cluster coordinated by two glutathione molecules and a conserved cysteine per each subunit of the homodimer, and transiently interacts with ISCA1 and ISCA2 to transfer the two [2Fe-2S]^2+^ clusters cargo to the apo ISCA1-ISCA2 hetero-complex via sequential molecular events [21,22]. Electrons required to couple the two received [2Fe-2S]^2+^ clusters are donated by FDX2 (Figure 1) [27], but no information is yet available on which is the specific electron acceptor. IBA57 has been shown to be required to assemble the [4Fe-4S]^2+^ cluster on ISCA1-ISCA2 complex, but its specific molecular function is still not clarified. IBA57 was shown to form a hetero-dimeric complex with ISCA2, but not with ISCA1, via bridging a [2Fe-2S] cluster [28,29]. Considering that the ISCA2-IBA57 complex can stabilize both reduced [2Fe-2S]^+^ and oxidized [2Fe-2S]^2+^ bound clusters [28], a possibility is that the [2Fe-2S] IBA57-ISCA2 heterodimeric complex is the entry point of the electrons donated by FDX2, but this still needs experimental evidence. The assembled [4Fe-4S]^2+^ cluster can then be inserted into apo recipient proteins, such as aconitase, without the requirement of further accessory proteins [6,30,31] or can be transferred to NFU1 or IND1 that then mediate the [4Fe-4S]^2+^ cluster insertion specifically into recipient proteins such as complex I, complex II and lipoyl synthase (LIAS) (Figure 1) [6,30,31]. While the molecular mechanism involving IND1 in the insertion of the [4Fe-4S]^2+^ cluster into complex I is not yet characterized, the insertion of the [4Fe-4S]^2+^ cluster into LIAS has been deeply investigated. Sequential protein–protein interactions involving ISCA1 and NFU1 have been shown to be responsible for the insertion of the [4Fe-4S] cluster into LIAS [32,33]. LIAS is a member of the radical S-adenosylmethionine (SAM) superfamily [34,35]. It catalyzes the final step of the biosynthesis of lipoyl cofactor [36,37,38] and binds two [4Fe-4S] clusters [39,40]: a [4Fe-4S] cluster (FeS_RS_), typical of all radical SAM enzymes, and a [4Fe-4S] cluster (FeS_aux_) that provides two sulfur atoms to the lipoyl cofactor [41,42]. It has been shown that the C-domain of NFU1 drives first [4Fe-4S]^2+^ cluster delivery from the ISCA1-ISCA2 complex, where the [4Fe-4S]^2+^ cluster is assembled, to a [4Fe-4S]^2+^ ISCA1-NFU1 intermediate complex, which then specifically directs the cluster into the FeS_RS_ site of LIAS [32,33]. According to this molecular function, NFU1 has been shown to form two hetero-complexes with ISCA1 and LIAS [32,33]. These two complexes have been recently characterized at a molecular level, and it has been shown that, in both cases, only the C-domain of NFU1 is involved in the protein–protein interaction [32,33]. This domain therefore drives the assembled [4Fe-4S]^2+^ cluster from the ISCA1-ISCA2 complex to the final destination. BOLA3 has been implicated in the latter transfer step to LIAS (Figure 1) on the basis of clinical phenotypes of patients with pathogenic variants in the *BOLA3* gene similar to those of patients with pathogenic variants in the *NFU1* gene [43]. However, how BOLA3 contributes to this process is yet not defined. Indeed, in vitro studies showed that NFU1 and BOLA3 do not interact with each other in various experimental conditions, i.e., BOLA3 with either apo or [4Fe-4S]^2+^ NFU1 [20,44]. The only well-defined partner of BOLA3 is GLRX5, which operates upstream in the [4Fe-4S] cluster maturation pathway as a [2Fe-2S] cluster donor. GLRX5 forms an apo hetero-dimeric complex with BOLA3 [45,46]. This apo complex is able to bridge a [2Fe-2S]^2+^ cluster between the two proteins being coordinated, on the GLRX5 side, by the conserved Cys67 and by the cysteine of a GLRX5-bound glutathione (GSH) molecule, and on the BOLA3 side by the conserved Cys59 and His96. This holo-complex has been shown in vitro to function in [2Fe-2S] cluster trafficking in the mitochondrial iron–sulfur protein biogenesis. The [2Fe-2S]^2+^ BOLA3-GLRX5 complex was shown indeed to transfer the cluster to both apo human ferredoxins FDX1 and FDX2 with rate constants comparable to other cluster donors to FDX proteins [20], as well as to transfer its cluster to apo NFU1 to form a [4Fe-4S]^2+^ NFU1 dimer [44]. However, considering that the yeast homologue of human BOLA3 was shown not to be required for the maturation of mitochondrial [2Fe-2S] proteins [46], the cluster transfer to FDXs is very likely not physiologically relevant. On the other hand, cluster transfer and assembly from [2Fe-2S]^2+^ GLRX5-BOLA3 to NFU1 was proposed to be alternative to the pathway involving the [4Fe-4S] cluster transfer from the ISCA1-ISCA2 complex to NFU1, being exclusively activated under oxidative cellular conditions [44] (Figure 1).

## 3. Mutations on Components Maturing ISC Proteins Cause Severe Congenital Diseases

Mutations in GLRX5, which connects the first step of the ISC machinery to its last step, as well as mutations in the accessory proteins, which are involved in the late-acting step of the ISC machinery devoted to mitochondrial [4Fe-4S]-binding protein biogenesis, cause different Fe-S cluster-related diseases, such as sideroblastic anemia, muscle myopathy, multiple mitochondrial disfunction syndromes 1 to 5, and complex I deficiency [23,30,47,48,49]. All of the aforementioned diseases are associated with severe, often lethal outcomes due to defects in mitochondrial [4Fe-4S] proteins, documenting the importance of these late-acting accessory proteins for cell viability. Here, below, we report a description of all pathogenic mutations identified up to now in GLRX5 and in the late-acting accessory proteins, and we describe, from a structural point of view, the effects that these mutations can have on the mutated proteins and on impairing their protein–protein interaction networks.

### 3.1. Structural Aspects of Pathogenic Missense Mutations in GLRX5, a Protein Involved in a Rare Form of Congenital Sideroblastic Anemia

Mutations in *GLXR5* gene have been associated so far with two different phenotypes, i.e., the variant nonketotic hyperglycinemia (NKH, MIM# 605899) [50] and the congenital sideroblastic anemia (SIDBA3, MIM#616860) [51]. NKH was associated with the non-sense p.K51del variant of GLRX5 [50]. On the contrary, missense mutations were found in patients affected by SIDBA3. Congenital sideroblastic anemia comprises a heterogeneous group of genetic disorders, characterized by reduced heme synthesis, mitochondrial iron overload and the presence of ringed sideroblasts [24]. In total, four pathogenic missense mutations (two pairs of heterologous pathogenic missense mutations) were identified in *GLRX5* gene (Table 1). Two heterozygous missense mutations were found in the *GLRX5* gene of a Chinese patient showing SIDBA3: Lys101Gln and Leu148Ser substitution in the GLRX5 protein [51]. Lys101 in GLRX5 protein is highly conserved from yeast to humans [51], whereas Leu148 is less conserved. Decreased holo IRP1 protein levels were observed in peripheral blood mononuclear cells (PBMCs), and, consequently, decreased activity of cytosolic aconitase was evident in PBMCs, together with increased expression of Transferrin receptor 1 (TfR1) protein and reduced expression of H-ferritin protein. Decreased ferrochelatase (FECH) level suggested mitochondrial Fe-S biogenesis impairment in PBMCs of the patient [51]. Daher et al. identified in a 14-year-old girl with SIDBA3, featuring heterozygous mutations in the *GLRX5* gene (Cys69Tyr and Met128Lys, Table 1). Functional studies of these variants were not performed, but studies in patient lymphoblastoid cells showed decreased activity in several Fe-S containing enzymes, including mitochondrial respiratory chain complexes I and mitochondrial aconitase (mACO), and in heme-containing enzymes, such as respiratory chain Complex IV [52].

**Figure 2:**
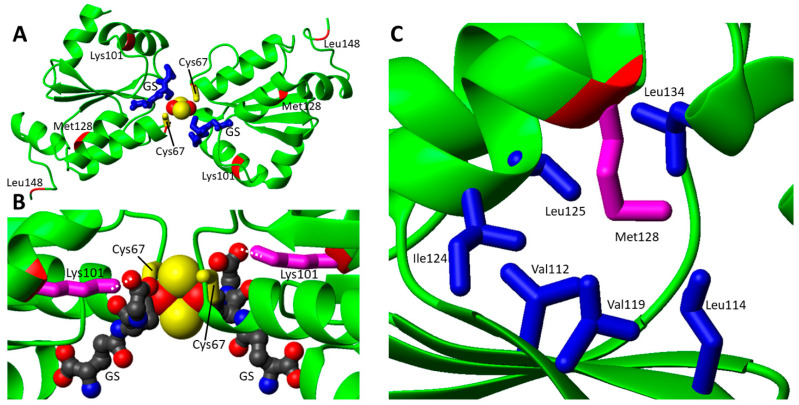
Pathogenic missense mutations mapped on the crystallographic structure of human GLRX5. (**A**) The backbone of the residues involved in pathogenic missense mutations of GLRX5 is shown in red on the ribbon structure of homodimeric [2Fe-2S] GLRX5 (PDB ID 2WUL). The sidechain of the conserved cluster coordinating Cys67 residue is shown in yellow. The iron and sulfur atoms of the [2Fe-2S] cluster are in red and yellow spheres, respectively; and glutathione (GS) ligand is shown in blue. (**B**) The electrostatic interaction established between the sidechain of Lys101 (in magenta) and the carboxylate group of the glutathione cluster ligand, shown in ball and stick mode, is shown as a dotted line. (**C**) The sidechains of the hydrophobic residues (in blue) interacting with the sidechain of Met128 (in magenta) are shown.

In order to analyze the effects of the pathogenic missense mutations from a structural point of view, two GLRX5 PDB entries (2WUL and 2MMZ) have been considered: the first is the crystallographic structure of the holo form, i.e., the protein in a homo-dimeric state in complex with a [2Fe-2S]^2+^ cluster and two glutathione molecules [53], while the second is a solution NMR structure of the monomeric apo protein [22]. The four identified missense pathogenic mutations were here mapped on the dimeric [2Fe-2S]^2+^ GLRX5 structure (Figure 2A). The two pathogenic mutations Lys101Gln and Cys67Tyr appears to have a major structural effect: the first one involves the disruption of the electrostatic interaction between the carboxylate group of glutathione and the ε-amino group of Lys101 sidechain in both [2Fe-2S] homo-dimeric GLRX5 and hetero-dimeric GLRX5-BOLA3 complexes (Figure 2 and Figure 4B). The second one is even more critical since it affects the coordination of the [2Fe-2S] cluster to GLRX5, as Cys67 is involved in iron coordination in both [2Fe-2S] homo-dimeric GLRX5 and hetero-dimeric GLRX5-BOLA3 complexes (Figure 2A,B and Figure 4B). The Met128Lys mutation involves a methionine that points to the interior in both apo and holo structures establishing hydrophobic contacts with nearby hydrophobic residues (Figure 2C). Thus, it is likely that the introduction of a charged Lys residue significantly impacts on these hydrophobic interaction patterns, thus destabilizing the structure of GLRX5. Mutation Leu148Ser does not provide clear structural evidence for an impairment of the protein function: it is located at the C-terminus in a mobile region with very poor electron density in 2WUL and distant from GSH and the cluster binding site (Figure 2A); likewise, the mutation is situated in a very mobile region also in the apo GLRX5 structure.

The pathogenicity of both pairs of mutations might therefore arise from a reduced [2Fe-2S] cluster chaperone activity of GLRX5 in mitochondria, having severe consequences on the efficiency of the ISC machinery and on the maturation of mitochondrial and extra-mitochondrial Fe-S proteins, the latter requiring both the mitochondrial and the cytosolic Fe-S cluster assembly machineries [7]. GLRX5 deficiency has been also linked to the impairment of heme biosynthesis and to the depletion of cytosolic iron [54], this possibly explaining the effects observed on Complex IV in the presence of pathogenic GLRX5 mutations causing SA [51,52].

### 3.2. Pathogenic Missense Mutations in the Late Acting Accessory Proteins of the ISC Machinery

Multiple Mitochondrial Dysfunction Syndromes (MMDS) types 1 to 5 are a group of rare but severe autosomal recessive diseases, caused by variants in the genes encoding for NFU1, BOLA3, IBA57, ISCA2 and ISCA1 proteins, respectively. The hallmark of these diseases is a decreased energy metabolism, which results in defects in neurologic development, muscle weakness, lactic acidosis, respiratory failure. Generally, the pathology manifests in early infancy and results in early death [23]. All of the MMDSs 1–5 have the main impact on enzymes whose functions rely on the presence of bound [4Fe-4S] clusters, such as LIAS and respiratory complexes I and II.

In addition to MMDS 1 to 5, two other genetic diseases are linked to genes of the late-acting step of the ISC machinery. The first is called Complex I deficiency, and is associated with mutations in the mitochondrial P-loop NTPase IND1, that is directly responsible for the maturation of the [4Fe-4S] cluster-containing subunits of respiratory chain complex I. A second is called Episodic mitochondrial myopathy with or without optic atrophy and reversible leukoencephalopathy (MEOAL), which has been linked to deficiency of FDX2. FDX2 is a protein that binds a [2Fe-2S] cluster that provides electrons to assemble the [4Fe-4S] cluster and is thus responsible for the maturation of all mitochondrial [4Fe-4S] target proteins.

#### 3.2.1. Structural Aspects of Pathogenic Missense Mutations of NFU1 Involved in MMDS1

Bi-allelic pathogenic variants in the *NFU1* gene cause MMDS type 1 (MMDS1, MIM#605711), clinically characterized by severe encephalopathy that manifests in the first year of life, with evidences of decreased energetic metabolism. Eleven missense (Table 1) and six non-sense disease-causing variants in NFU1 have been identified to date in patients affected by MMDS1. The metabolic profiles of these patients are characterized by a decreased activity of several mitochondrial proteins, such as pyruvate dehydrogenase (PDH), Glycine Cleavage System (GCS) and respiratory chain complexes. PDH and GCS decreased activity was due to lipoic acid synthesis impairment.

**Figure 3:**
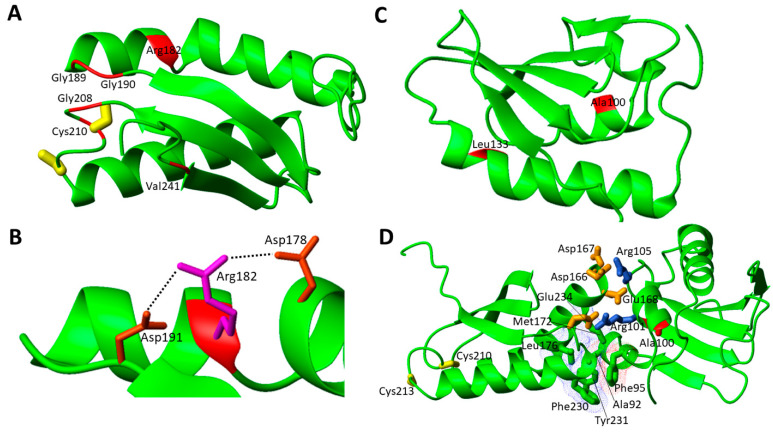
Pathogenic missense mutations mapped on the solution structures of the isolated N- and C-terminal domains of human NFU1 and on a structural docking model describing their interaction. (**A**–**C**) The backbone of the residues involved in pathogenic missense mutations in the C-terminal (**A**) and N-terminal (**C**) domains of NFU1 is shown in red on the ribbon structures of these domains (PDB ID 2LTM and 2M5O, respectively). The sidechains of the two conserved cluster coordinating Cys residues on the C-terminal domain of NFU1 are shown in yellow. (**B**) The electrostatic interactions established by sidechain of Arg182 (magenta) and the sidechains of the surrounding Asp residues (in orange) are shown as dotted black lines. (**D**) Docking structural model of the interaction between the N-terminal and C-terminal domains of NFU1 is shown. Ala100 of the N-terminal domain (in red) is close to Arg101 and Arg 105 (both in blue), which are in turn involved in electrostatic interactions with Glu and Asp residues of the C-terminal domain (all in orange). Interacting hydrophobic patches between the two domains (Van der Waals surfaces are in blue and red for the C- and N-domains, respectively), which involve both aromatic and aliphatic residues (sidechains are in green) and are close in space to Ala100 (sidechain is in red), are also shown.

The structural characterization of apo NFU1 showed that the apo form is monomeric in solution and adopts a dumbbell-shaped structure with well-structured N- and C-domains connected by a linker [54]. It has been also shown that NFU1 binds a [4Fe-4S]^2+^ cluster, which induces the formation of a homo-dimer through the bridging [4Fe-4S]^2+^ cluster, coordinated by two cysteines of the conserved CXXC motif of each of the two C-domains [44]. Moreover, the presence of an equilibrium between the [4Fe-4S]^2+^ NFU1 dimer with the cluster coordinated by the Cys residues of the two CXXC motifs, and a [4Fe-4S]^2+^ NFU1 dimer where a Cys ligand of the CXXC motif is replaced by a S-donor small molecule ligand, such as GSH or DTT, was observed [44]. In order to analyze the effects of the pathogenic missense mutations on the protein structure, we have considered the solution NMR structures of the single N- and C-domains of NFU1 (2LTM and 2M5O, respectively), since no structure of the full-length protein is yet available. The missense pathogenic mutations are mainly located on the C-domain of NFU1 in the surrounding of the cluster binding CXXC motif (7 out of 11 mutations, Figure 3A).

The c.545G > A (p.Arg182Gln) pathogenic missense mutation was found in two cases. One is in homozygosity, where no mature protein could be detected in fibroblast mitochondria as a consequence of defective mRNA splicing caused by this mutation [43]. It was suggested that the mutant protein might undergo degradation [43]. Another patient carried both the c.622G > T (p.Gly208Cys) and the c.545G > A (p.Arg182Gln) heterozygous mutations in *NFU1* gene. Metabolic findings were high blood lactate and urine glutaric acid levels, but no information about the impairing of any mitochondrial Fe-S protein is available [59]. Arg182 is also involved in another pathogenic missense mutation, i.e., c.544C > T (p.Arg182Trp). This mutation is found in heterozygosity with the Gly208Cys missense mutation [63]. It was shown that in the processed mRNA, only the c.544C > T gene mutation was detectable in the patient’s fibroblasts, suggesting that the protein can also be detected in fibroblast mitochondria at variance with the c.545G > A missense mutation [63]. Both Arg182 mutations appear to be critical from the chemical point of view since they involve the disappearance of a charged residue. Two Asp residues are located nearby to Arg182, and this charge loss in the pathogenic mutants can thus perturb these electrostatic interactions, destabilizing the overall structure as well as protein–protein recognition (Figure 3B). Arg182 is solvent exposed and is located on the helix of the C-domain of NFU1 involved in the complex formation with both ISCA1 and LIAS [32,33]. Its mutation into Gln or Trp might negatively affect the protein–protein recognition between the C-domain of NFU1 and both ISCA1 and LIAS and the [4Fe-4S] cluster transfer to LIAS. Thus, the two pathogenic mutations, destabilizing the protein complex, might determine a significant decrease in the cellular content of mature LIAS, and consequently a defective lipoylation of the acid-dependent 2-oxoacid dehydrogenases and of the glycine cleavage system. This model is confirmed by the high deficiency of those enzymes requiring lipoate in patients carrying mutations on Arg182 [43,59,63].

Gly208Cys was found both in homozygous and heterozygous states. This variant was specifically linked to pulmonary arterial hypertension [55]. The mutation modifies the native Fe-S binding CXXC pattern of NFU1 into CXCXXC. The mutation does not significantly impact on the protein stability and leads to a slight decrease in alpha helix and beta sheet content with a corresponding increase in the random coil part [97]. Differential scanning calorimetry and analytical ultracentrifugation experiments also suggested an increased tendency of the protein to oligomerize, specifically to the dimeric form. From in vitro cluster transfer experiments, it was argued that Gly208Cys NFU1 is not able to receive a cluster in vivo and would therefore be unable to transfer a cluster to downstream partners [97].

Another missense pathogenic mutation found in the C-domain is Gly189Arg in homozygosis, which is the second most frequent variant (5 patients). It has been suggested that this variant is associated with a milder progressive disease [63], as, out of the five patients, one survived till the age of 41 months [57], and another one is still alive in its adulthood [98]. The latter is carrying a second heterozygous variant (Cys210Phe) and showed an atypical clinical phenotype, with a later onset of the first neurological manifestation of the disease and partial recovery. The patient showed decreased activity of PDH in the fibroblasts. The Gly189Arg mutation is located close to the cluster binding site and thus the introduction of a charged and bulky residue might drastically compromise [4Fe-4S] cluster binding. This mutation has been characterized in a quite recent paper [99]; it has been shown that the mutation induces structural changes that increase flexibility, decrease stability, and alter the monomer–dimer equilibrium toward the monomeric form, thus impairing the ability of the Gly189Arg moieties to receive the cluster from physiologically relevant partners.

Next to the Gly189Arg mutation, there is one of the same nature, i.e., Gly190Arg, that is in heterozygosity with Gly189Arg [63]. Both amino acids are highly conserved, and their mutation was predicted to have a major impact on protein function by several programs (PolyPhen2, SIFT, MutationTaster) [63]. As in the case of Gly189Arg, this mutation implies the non-native presence of a charged and bulky residue, potentially negatively affecting [4Fe-4S] cluster binding ability.

Cys210Phe was found in heterozygosity with i) Gly208Cys mutation [58], with the patient showing the same clinical features as those usually found in patients with the Gly208Cys mutation only, and moderately reduced activity of respiratory chain complex IV, and ii) Gly189Arg [62]. In the latter case, Western blot analysis performed on patient’s fibroblasts showed no reduction in the NFU1 level, but a partial reduction of that of the subunits of complex II, SDHA (70 kDa), and SDHB (30 kDa), whereas the levels of the subunits of the other respiratory chain complexes are not affected [62]. Cys210 is one coordinating ligand of the [4Fe-4S]^2+^ cluster in the NFU1 homodimer [44,54]. Therefore, this pathogenic mutation abolishes the ability of NFU1 to bind the [4Fe-4S]^2+^ cluster.

The missense pathogenic mutation Val241Phe was found in heterozygosis with exon 4 deletion of the *NFU1* gene. Val241 is located close to the C-terminus of the C-domain of NFU1 at the end of the last β-strand and might therefore play a role in the C-domain structural conformation and stability [65] as a consequence of the introduction of a bulkier aromatic residue with respect to the original one.

There are only four pathogenic missense mutations in the N-domain of NFU1. At variance with the C-domain, the functional role of the N-domain of NFU1 in the maturation of mitochondrial [4Fe-4S] proteins is still debated, as it does not directly contribute to the interactions with LIAS and ISCA1. The structural effect of these mutations are mapped and analyzed on the solution NMR structure of the single N-domain of NFU1 (Figure 3C).

Arg21Pro is in heterozygosity with Gly208Cys. Biochemical analysis showed clear complex II deficiency, and decreased activity of complex I in liver tissues [63]. Phe60Cys was found in homozygosis in a patient showing clinical features typical of MMSD1, but normal α-ketoglutarate dehydrogenase (α-KGDHc) activity and protein lipoylation [58]. Arg21 and Phe60 are located at the N-terminal unstructured segment of NFU1, which constitutes the mitochondrial targeting sequence composed by the first 58 N-terminal residues and, once processed by mitochondrial processing peptidases, produces the mature form of NFU1 with a molecular mass of ~22 kDa [100]. We can predict that these pathogenic mutations do not affect the structural feature of NFU1 as well as its functional interaction network, but rather they might be crucial in the import in the mitochondrial matrix.

Ala100Gly was found in heterozygosity with the Leu133Pro missense mutation. No biochemical analysis has been reported. Both residues are located in the core of the domain and form hydrophobic interactions with nearby non-polar residues. In this respect, the Ala-to-Gly mutation should have a minor impact on the local folding properties. However, this mutation might be critical in the interaction between the N- and C-domains of NFU1. Indeed, Ala100 is located in a region involved in the interaction between the two domains [44]. A docking structural model of the two domains [44] shows that they interact through a hydrophobic patch and a charged patch, and therefore, the Ala100Gly mutation might negatively affect this inter-domain interaction in such a way to impair the functional role of the N-domain (Figure 3D). On the contrary, the Leu133Pro missense mutation could be critical for the structural stability of the N-domain since the presence of a Pro can break the α-helix where Leu 133 is located.

#### 3.2.2. Structural Aspects of Pathogenic Missense Mutations of BOLA3 Involved in MMDS2

**Figure 4:**
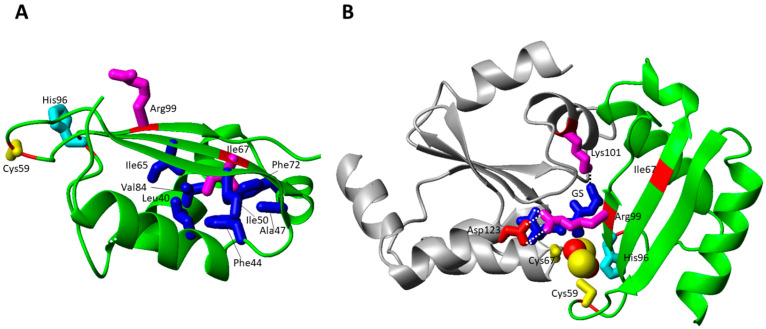
Pathogenic missense mutations mapped on the structures of human BOLA3 and [2Fe-2S] GLRX5-BOLA3 complex. (**A**) The backbone of the residues involved in pathogenic missense mutations of BOLA3 is colored in red on the solution structure of apo BOLA3 (PDB ID 2NCL), and the sidechains of the conserved cluster coordinating Cys59 and His96 residues are shown in yellow and cyan, respectively. The sidechains of Arg99 and Ile67 residues involved in pathogenic missense mutations are shown in magenta. The sidechains of the hydrophobic residues interacting with Ile67 are shown in blue. (**B**) The ribbon diagram of the structural model of [2Fe-2S] GLRX5-BOLA3 available from a data-driven docking approach [45] is shown. GLRX5 is in grey and BOLA3 is in green. The sidechains of the two Cys ligands and of the His ligand are shown as yellow and cyan stick, respectively; the iron and sulfur atoms of the [2Fe-2S] cluster are in red and yellow spheres, respectively; and GS cluster ligand is shown in blue. The sidechains of the residues involved in pathogenic missense mutations at the interface between BOLA3 and GLRX5 are shown in magenta. Electrostatic interactions involving these residues are shown as dotted lines.

Bi-allelic variants in BOLA3 cause MMDS type 2 with hyperglycinemia (MMDS2; MIM#614299), typically characterized by infantile encephalopathy, leukodystrophy, lactic acidosis, nonketotic hyperglycinemia and death in early childhood [5,7,11]. Four missense (Table 1) and four non-sense disease-causing variants in BOLA3 have been identified to date in patients affected by MMDS2 [43,50,66,67,68,69,70,72]. The four missense pathogenic mutations have been mapped on the solution NMR structure of BOLA3 [46] as well as on a structural model of the hetero-dimeric complex formed by BOLA3 and GLRX5 with a bridging [2Fe-2S]^2+^ cluster (Figure 4) [45].

The Ile67Asn pathogenic mutation was found in homozygosis in two siblings, exhibiting severe neonatal lactic acidosis, hypotonia, and intractable cardiomyopathy; high pyruvate, lactate and glycine levels were found in both of them. Both patients died within the first months of life [66]. This mutation is reported to be critical as highlighted by a recent paper [101], which showed the impairment of BOLA3 to bind its physiological partner GLRX5 in the apo form as well as the inability to form the [2Fe-2S]^2+^ complex with GLRX5. However, this mutation does not have dramatic structural effects on BOLA3 as well as on the BOLA3-GLRX5 hetero-complex, as this residue is located away from the cluster binding site and from the interacting surfaces with the protein partner (Figure 4B). Ile67 is, indeed, located in the middle of the β-sheet being involved in hydrophobic interactions in the core of the protein (Figure 4A). Thus, these structural features do not clearly explain the observed behavior of this mutant in preventing apo complex formation with GLRX5 as well as in abolishing cluster binding properties of the BOLA3-GLRX5 complex.

The His96Arg homozygous mutation of BOLA3, which was found in the Japanese population, caused severe lactic acidosis and combined respiratory chain complex deficiencies (decreased complex II activity), multiple organ failure, and hypertrophic cardiomyopathy seizures. Metabolic findings were high lactate and glycine levels. The majority of the patients died in the first month of life [68,69,70]. The mutation is on a fully conserved residue, which characterizes the BOLA protein family [102]. Similarly, the Cys59Tyr mutation involves a highly conserved residue typically conserved in the BOLA3 family. This pathogenic mutation was found in heterozygosis with c.136C > T, p.Arg46 * deletion, and showed a milder phenotype with respect to His96Arg homozygous mutations [72].

The two His96Arg and Cys59Tyr pathogenic mutations involve the BOLA3 cluster ligands in the [2Fe-2S]^2+^ BOLA3-GLRX5 complex, i.e., Cys59 and His96 (Figure 4B). Both mutations abrogate the coordination ability of the residues in these two positions, thus possibly resulting in a tri-coordinated [2Fe-2S]^2+^ cluster in the hetero-complex. This model is supported by a mutagenic study performed on the BOLA3-GLRX5 complex and on a homologous complex, formed by GLRX5 and BOLA1, that also coordinates a [2Fe-2S] cluster with the same invariant C-terminal His of BOLA3, that is His102 in BOLA1. Both His96Ala BOLA3 and His102Ala BOLA1 mutations showed that the [2Fe-2S] cluster remains bound to the mutated hetero-complex, but demonstrated that the invariant His plays a crucial role in forming a [2Fe-2S] hetero-complex in a unique cluster bound conformation as well as in stabilizing the cluster with respect to its reduction [45]. These effects are likely physiologically relevant as the invariant C-terminal His, once mutated to Ala, in the yeast BOLA1 and BOLA3 homologues was shown to be important for the respiratory growth of cells [46,103], suggesting that the pathogenic His96Arg mutation might have a negative effect on the so far proposed function of the [2Fe-2S] BOLA3-GLRX5 complex as cluster donor to NFU1, as reported in an in vitro study [44].

For the Cys59Tyr mutation, a deep experimental study investigated the role of the mutation in both the formation of the apo BOLA3/GLRX5 hetero-complex and in the iron-sulfur cluster-binding properties of the hetero-complex [104]. From that study, it resulted that: (i) the mutation structurally perturbs the iron–sulfur cluster-binding region of BOLA3, but without abolishing the [2Fe-2S]^2+^ cluster-binding ability to the hetero-complex; (ii) Tyr 59 does not replace Cys 59 as an iron–sulfur cluster ligand; and (iii) the mutation promoted the formation of an aberrant apo BOLA3-GLRX5 complex. All these aspects allowed rationalizing the unique phenotype observed in MMDS2 caused by the Cys59Tyr mutation. The patient characterized by heterozygous variants in BOLA3, i.e., c.136C > T, p.Arg46 * and c.176G > A, p.Cys59Tyr, has a much milder phenotype with respect to homozygous c.136C > T (p.Arg46 *) variants [50,72]. The latter variants resulted as incompatible with life, and at variance with the heterozygous variants, which resulted in a complete clinical recovery after the age of 8. This suggested to us that the Cys59Tyr BOLA3 variant might maintain some functional activity and thus might compensate for, at least partially, the effect of the other p.Arg46 * BOLA3 heterozygous variant. In conclusion, the available structural data indicated that the His96Arg mutation affects the complex and cluster binding stability much more dramatically than the Cys59Tyr mutation, in agreement with the latter mutation being compatible with life while the His96Arg mutation results as lethal.

The Arg99Trp pathogenic mutation, found in homozygosis in two patients, showed milder phenotypes, one of them still being alive at the age of 12, without any sign of cardiomyopathy [71]. The sidechain of this residue is solvent exposed and is located on the last β-strand (Figure 4A). In the [2Fe-2S] BOLA3-GLRX5 complex, it points towards the GLRX5 subunit forming an electrostatic interaction with Asp123 of GLRX5 (Figure 4B). The GSH cluster ligand is also contributing to the structural stabilization of this region through electrostatic interactions of its carboxylate and amine groups with both the charged Arg99 and Asp123 residues (Figure 4B). Thus, the mutation of Arg to Trp totally disrupts all these stabilizing interactions, possibly negatively affecting the cluster binding property of GSH and hetero-complex formation.

#### 3.2.3. Structural Aspects of Pathogenic Missense Mutations of IBA57 Involved in MMDS3

Several bi-allelic variants in the *IBA57* gene cause MMDS type 3 (MMSD3, MIM# 615330). Overall, 36 pathogenic variants of IBA57 were reported so far, with a broad phenotypic spectrum ranging from severe intellectual disability to adolescent-onset spastic paraplegia. Several patients carrying IBA57 mutations survived until adulthood. The most common clinical feature was psychomotor regression with progressive spasticity. Frequent metabolic findings were hyperlactatemia and in some cases hyperglycinemia. The activity of the respiratory complexes I, II, and IV were often impaired, with an associated low level of lipoylated proteins in the fibroblasts. The specific genetic, clinical and biochemical effects that the identified missense mutations determine are described hereafter.

The c.706C > T (Pro236Ser) mutation was found in homozygosity [73] and in heterozygosity with the Tyr219Cys mutation [71]. Homozigous c.706C > T mutation resulting in Pro236Ser mutation on IBA57 was found in four unrelated patients, all exhibiting combined or isolated defects on complexes I and II [73]. Different clinical features were observed, ranging from fatal infantile onset to severe, acute psychomotor regression after the first year of life, with partial recovery in at least one of the patients [73]. High lactate and pyruvate levels were commonly observed in blood and cerebrospinal fluid (CSF) of the patients [73]. One case showed significantly decreased expression of mACO, together with slightly overexpressed ISCA1 [73]. The same mutation found in heterozygosity with the c.656A > G (p.Tyr219Cys) mutation of the *IBA57* gene was diagnosed in only one patient with leukoencephalopathy, displaying a milder phenotype and being still alive at the age of 2. No biochemical or metabolic analyses of the patient are reported.

The Gln314Pro homozygous mutation (c.941A > C) was found in two siblings from consanguineous parents [74]. The two patients, who died few days after birth, had increased glycine levels in CSF and serum, and a combined respiratory chain deficiency of complex I and II. Defects in lipoate-containing enzymes and mACO were also observed. Biochemical studies demonstrated that IBA57 carrying the Gln314Pro mutation is rapidly degraded in HeLa cells [74].

The Tyr96His mutation of IBA57 was found in a high number of patients, in heterozygosity with several missense (Gly63Asp, Thr106Ala, Gly252Cys) and non-missense (c.697C > T, Arg223 *; p.307C > T, Gln103 *; c.22C > T, Arg8 *; c.522_523del, Leu175Alafs; c.589_590del, Arg197Alafs; c.1053G > A, Trp351 *; c.589_590del, Arg197Alafs) mutations [75,76,105]. Most cases showed a stabilized or improved pattern after an acute or subacute onset, experiencing, in general, severe motor handicap and delay in communication, but all the patients were still alive at various ages [76,77,105]. No biochemical or metabolic characterizations are reported.

The Thr106Ala IBA57 mutation was found in heterozygosity, in addition to Tyr96His, also with several other missense mutations, such as Asn246Lys [58] and Val253Leu [58]. The Thr106Ala/Asn246Lys variant was found in a female patient who died at the age of 2.5 months. For this patient, impaired activity and assembly of complex II was observed [58]. The combined heterozygous p.Thr106Ala (c.316A > G) and p.Val253Leu (c.757G > C) variant was found in a patient who developed a severe phenotype after a normal development and a healthy period, followed by sudden death [73]. Biochemical analysis showed reduction of the respiratory chain complex II activity in fibroblasts mitochondria [73].

The p.Asn246Lys mutation was also found in heterozygosity with the p.Arg268Cys mutation (c. 738 C > G + c.802 C > T) [71]. These mutants were described in a study performed by Bindu et al. on a group of 14 patients, all exhibiting mitochondrial leukoencephalopathy with similar clinical features, ranging from encephalopathy, to febrile illness preceding the onset, neurological dysfunction and a subsequent stable rather than a progressive course [71]. No biochemical analysis was performed on the patients carrying the mutations.

The heterozygous c.335T > G and c.437G > C mutations in *IBA57* gene, leading, respectively, to Leu112Trp and Arg146Pro mutations on the IBA57 protein were found in a patient who died at the age of 11 months. Impaired activity and assembly of complex II was reported for this patient [58]. A second mutation affecting Leu112 of IBA57 was found in heterozygosity with a non-missense mutation (c.588dup, p.Arg197Alafs) in two siblings. Specifically, Leu112 was mutated into Ser (c.335T > C) [77]. The first patient carrying the Leu112Ser and the Arg197Alafs mutations was still alive at the age of 29 [77], showing a milder phenotype, with infantile-onset optic atrophy, than that of previously reported cases of IBA57 mutations. The second patient carrying these mutations is a 19-year-old male and the younger brother of the first described patient. He developed spastic paraparesis in addition to infantile-onset optic atrophy [77]. Biochemical analysis showed, for both patients, normal levels of activity for respiratory chain complex enzymes.

Arg146 was also found homozygously mutated into Trp (c.436C > T, p.Arg146Trp) in a young male patient. The patient, who died at the age of 17 months, had, at the age of six months, signs of motor regression due to progressive hypotonia and muscle weakness. Metabolic investigations showed a mild increase in lactate and glycine concentrations in CSF and blood [79]. Biochemical studies revealed decreased protein levels and activity of respiratory chain complex I and II enzymes [79]. Moreover, analysis of the lipoylation of the E2 subunits of PDH and alpha-ketoglutarate dehydrogenase (αKGDH) revealed a severe decrease of lipoic acid levels for both enzymes, overall suggesting Fe-S cluster biosynthesis defects [79].

The Tyr108Ser mutation was found in heterozygosity with either the p.Gln314 * or the pCys50 * mutations of IBA57 protein in a group of three patients [78]. Protein expression analysis revealed significant decrease in IBA57 protein expression in myoblasts and fibroblasts. In addition, the NFU1 protein expression was also decreased. As a consequence, immunoblotting revealed reduced expression of the SDHB subunit of complex II, and of LIAS. Levels of lipoylated pyruvate dehydrogenase complex-E2 and α-ketoglutarate dehydrogenase-E2 were also reduced [78].

The heterozygous c.586T > G (p.Trp196Gly) and c.686C > T (p.Pro229Leu) variant was found in a patient that showed clinical signs since birth and developed a severe phenotype followed by sudden death [73]. Biochemical analysis pointed out a strong impairment of the respiratory chain complex II, a reduction of the levels and of the activity of most enzymes containing [4Fe-4S], such as mACO, as well as of enzymes containing lipoic acid, such as the α-ketoglutarate dehydrogenase complex (KGDHc), pyruvic dehydrogenase complex (PDHc) and α-branched-chain keto acid dehydrogenase complex (BCKDHc) enzymes [73].

The c.313C > T (p.Arg105Trp) mutation was found in heterozygosity with the non-missense c.87ins_GCCCAAGGTGC (p.Arg30Alafs) mutation. The patient exhibited severe disease onset after a normal psychomotor development and a good health period. Biochemical analysis showed a reduction of the respiratory chain complex II activity in fibroblasts mitochondria of the patient, and no significant reduction of the activity of KGDHc and BCKDHc, while the activity of PDHc was moderately reduced. Treatment of the patient with riboflavin might have improved the stability and activity of those apo-flavoenzymes that contain FAD in their active sites [73,106]. Interestingly, ISCA1 protein resulted slightly overexpressed [73].

A combined heterozygous c.236C > T (p.Pro79Leu) and c.307C > T (p.Gln103 *) variant of IBA57 was found in a 9-months-old patient with a mild phenotype and stabilized/improved pattern [76]. No biochemical analysis is reported.

The Asp234Gly and Ile261Thr heterozygous mutations of IBA57 were found in a patient whose case is described in a study involving 11 patients all characterized by leukodystrophy attributable to IBA57 mutations [75,76]. All patients survived and gradually improved. No specific data are reported for the patient carrying the above-mentioned mutations.

The Asp129Val and Glu244Ala combined heterozygous mutations were found associated with a severe phenotype, found in a 7-year-old Japanese girl with developmental regression, spastic quadriplegia, and seizures, which progressed rapidly after a febrile illness [77]. Moreover, brain MRI showed progressive, significant loss of white matter volume. Lactate and pyruvate levels were normal in blood and cerebrospinal fluid [77].

A combined heterozygous IBA57 variant with c.580A > G (p.Met194Val) and c.286T > C (p.Tyr96His) was found in a Chinese patient, who experienced acute and progressive psychomotor regression with hypertonia, limbs rigid, and trunk opisthotonos [80], and with a brain MRI profile of diffuse vacuolar leukoencephalopathy [80]. The patient died two months after the diagnosis [80]. No metabolic and biochemical analysis were performed.

Considering the large number of pathogenic missense mutations found in IBA57, i.e., twenty-four mutations (Table 1), we have grouped them on the basis of their solvent accessibility and analyzed their impact on the structures of IBA57 [28,107] and of the [2Fe-2S] IBA57-ISCA2 complex [29]. The solvent accessibility was obtained with the program NACCESS applied to the available crystallographic structure of IBA57, considering a residue as solvent-exposed when the relative solvent accessibility of the side-chain is over 40%.

**Figure 5:**
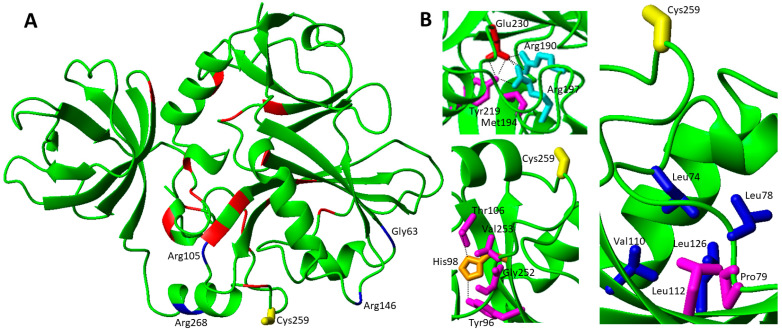
Pathogenic missense mutations mapped on the crystallographic structure of human IBA57. (**A**) The backbones of the buried and solvent-accessible residues involved in pathogenic missense mutations of IBA57 are colored in red and blue, respectively, on the ribbon structure of IBA57 (PDB ID 6QE4). The sidechain of the conserved cluster coordinating Cys259 is shown in yellow. (**B**) The sidechains of the residues involved in pathogenic missense mutations are shown in magenta. Hydrogen bond interaction is shown as a dotted black line. The positively charged, negatively charged and hydrophobic residues interacting with the residues shown in magenta are shown in cyan, red and blue, respectively.

19 missense pathogenic mutations, i.e., Tyr219Cys, Met194Val, Tyr96His, Pro79Leu, Thr106Ala, Tyr108Ser, Leu112Ser, Leu112Trp, Asp129Val, Trp196Gly, Pro229Leu, Asp234Gly, Pro236Ser, Glu244Ala, Asn246Lys, Gly252Cys, Val253Leu, Ile261Thr and Gln314Pro, involve buried residues (Figure 5A). Specifically, Tyr219Cys have dramatic steric effects, causing a drastic decrease of the hindrance in a buried zone; in addition, the mutation disrupts an extended hydrogen bond interaction network involving the Tyr219 OH group (Figure 5B). The same hydrogen bond interaction network might be affected also in the Met194Val mutation. Indeed, the side-chain of Met 194 is very close to the OH group of Tyr219 (Figure 5B). Thus, the Met194Val mutation, that determines a shorter side-chain with the absence of a S-methyl group, might preclude optimal contacts, thus negatively affecting the nearby hydrogen bond interaction network involving the OH group of Tyr219. Tyr96His is buried in the core of the structure, with Tyr96 being involved in a hydrogen bond with His98 (Figure 5B); this mutation can thus significantly modify the hydrogen bond pattern in the core of the protein. Similarly, the Thr106Ala mutation is buried inside the protein and causes the disruption of a hydrogen bond with His98 (Figure 5B). Gly252 is in a loop region close to Tyr96 (Figure 5B). The Gly252Cys mutation can have relevant steric effects, since the bulkier side chain of Cys can disrupt an extensive hydrogen-bond network close to Gly252, which might be relevant for properly orienting the loop that contains the functional Cys 259. Similarly, the Val253Leu mutation just adds one carbon unit, but in a buried environment and this modification, and although at first glance it could appear not particularly relevant, it might be crucial because the functional Cys 259 is just five residues away (Figure 5B). Tyr108 is located in an interior β-strand and is fully surrounded by hydrophobic residues with no polar or charged surrounding residues. Thus, Tyr108Ser causes a great change in steric hindrance and hydrophobicity, structurally perturbing the contacts between the β-strand where Tyr108 is located and the facing helix. The Asp129Val mutation causes a change in a region that is largely polar and charged; in particular, Arg56 is close to Asp129. The introduction of a hydrophobic residue as Val can thus irreversibly perturb the structure of this region. Trp196Gly causes a large change in steric hindrance, which might perturb the extensive steric contacts that Trp196 establishes with both the charged and hydrophobic surrounding residues. The interior loop region, comprising residues 198-218 and which is sandwiched among the three domains of IBA57, contains several buried residues involved in pathogenic mutations, which are Pro229Leu, Asp234Gly, Pro236Ser, Glu244Ala and Asn246Lys. The Pro229Leu and Pro236Ser mutations can both negatively affect the backbone conformation of the loop. Similarly, the Asp234Gly mutation disrupts several hydrogen-bond interactions that the carboxylate group of the side-chain of Asp234 establishes with Arg223 and Tyr224. The Glu244Ala mutation causes a change in charge, disrupting the charge interaction between the carboxylate of Glu244 and the amine group of the side-chain of Lys277 located in a facing β-strand. Asn246Lys appears crucial as well: there is again a change in charge, a disruption of a hydrogen bond with Arg278 and a larger hindrance in a buried and quite crowded protein region. The Leu112Trp mutation is sterically significant since it occurs in a buried and packed region (Figure 5B) while the Leu112Ser mutation introduces a polar residue in a fully hydrophobic, buried environment. Leu112 is in close contact with Pro79, located in a loop facing the sidechain of Leu112 (Figure 5B). The Pro79Leu mutation affects a region relevant for the stability and function of IBA57. Indeed, the cluster coordinating Cys 259 of IBA57 is not so far from this mutation site (Figure 5B). Ile261Thr is a significant mutation, as it introduces polarity in a largely hydrophobic region that is very close to the cluster coordinating Cys 260. Thus, this mutation can structurally perturb a region crucial for the protein function (see also below). Finally, the Gln314Pro mutation shortens the β-strand where Gln314 is located as a consequence of the lack of backbone NH for Pro as well as it disrupts a stacking interaction between the amide side-chain of Gln314 and the indole moiety of Trp217, which caps Gln314 from a nearby interacting α-helix.

**Figure 6:**
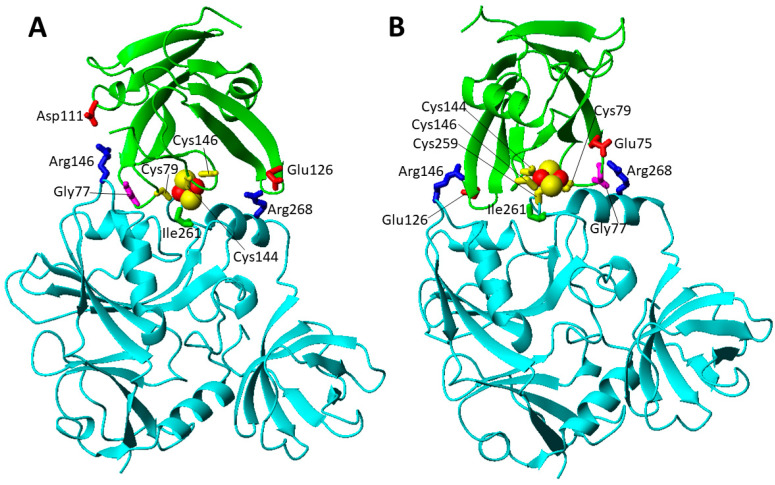
Effects of the pathogenic missense mutations on the heterodimeric [2Fe-2S] ISCA2-IBA57 complex. Ribbon diagrams of the two structural models of [2Fe-2S] ISCA2-IBA57 available from a data-driven docking approach [29] are shown in panels (**A**,**B**). IBA57 is in cyan and ISCA2 is in green. The sidechains of the four Cys ligands are shown as yellow stick, the iron and sulfur atoms of the [2Fe-2S] cluster are in red and yellow spheres, respectively. The side chains (involved in intermolecular electrostatic interactions) of Asp111 (red, ISCA2) and Arg146 (blue, IBA57) and of Glu126 (red, ISCA2) and Arg268 (blue, IBA57) in (**A**), and of Glu75 (red, ISCA2) and Arg268 (blue, IBA57) and Glu126 (red, ISCA2) and Arg146 (blue, IBA57) in (**B**) are shown as sticks. The backbone of Gly77 (magenta, ISCA2) and the side chain of Ile261 (green IBA57) are also shown as sticks on both models.

The missense pathogenic mutations involving highly solvent exposed IBA57 residues are five, i.e., Gly63Asp, Arg105Trp, Arg146Trp, Arg146Pro, and Arg268Cys (Figure 5A). Both Gly63 and Arg105 residue are located ~17 Å apart from the cluster coordinating Cys 259 at the end of two different β-strands that are part of the same β-sheet (Figure 5A). It is possible that both the Gly63Asp and Arg105Trp mutations, which introduce residues with very different chemical properties, perturb the structural boundaries of the β-sheet. On the contrary, the other solvent exposed missense mutations can negatively affect the formation of the heterodimeric [2Fe-2S] cluster-mediated complex of IBA57 with ISCA2 through a bridging [2Fe-2S] cluster, coordinated by the three conserved cysteines of ISCA2 and the conserved cysteine of IBA57 [29]. The two available low-resolution structural models of this complex (model A and B, hereafter) [29] have been here exploited to analyze the possible effects of the Arg 146 and Arg 268 pathogenic mutations on the complex formation and cluster binding. Arg 146 and Arg 268 are largely solvent exposed and involved in electrostatic interactions with Asp 111 and Glu 126 of ISCA2 in model A (Figure 6A) and with Glu126 and Glu75 of ISCA2 in model B (Figure 6B), respectively. Thus, mutation of these Arg residues into Cys, Pro and Trp (Arg146Trp, Arg268Cys, Arg146Pro) abolishes these electrostatic interactions, possibly destabilizing the cluster-mediated complex formation. This is indeed what has been experimentally observed in vitro for the Arg146Trp mutant that impairs the complex formation and cluster binding between IBA57 and ISCA2 [29]. This proposed effect also agrees with the finding that the amino acid mutation Arg146Trp in IBA57 impairs its function in mitochondrial [4Fe-4S] protein biogenesis without affecting IBA57 protein stability in vivo [79]. In addition, all these aspects support the proposal that the [2Fe-2S] ISCA2-IBA57 hetero-complex is a physiologically relevant species playing a role in mitochondrial [4Fe-4S] protein maturation. Among the IBA57 residues potentially affecting [2Fe-2S] cluster binding and the resulting complex formation, there is also the Ile261 residue that is next to the Cys259 cluster ligand and is spatially close to the cluster in the [2Fe-2S] IBA57-ISCA2 complex (Figure 6). Thus, the IBA57 pathogenic mutation Ile261Thr can potentially destabilize the [2Fe-2S] cluster binding, by disrupting the cluster-mediated complex.

#### 3.2.4. Structural Aspects of Pathogenic Missense Mutations of ISCA2 Involved in MMDS4

**Figure 7:**
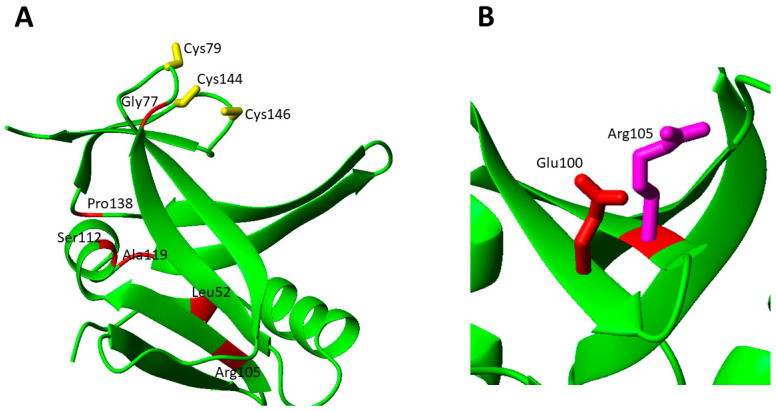
Pathogenic missense mutations mapped on the structural model of human ISCA2 obtained by AlphaFold2. (**A**) The backbone of the residues involved in pathogenic missense mutations of ISCA2 is colored in red on the ribbon structure of monomeric apo ISCA2, and the sidechains of the three conserved cluster coordinating Cys residues are shown in yellow. (**B**) The electrostatic interaction established between the negatively charged carboxylate of the sidechain of Glu100 (in red) and the positively charged guanidinium group of the sidechain of Arg105 (in magenta) is shown.

Bi-allelic variants of the *ISCA2* gene cause MMDS type 4 (MMSD4, MIM#616370). The most common biochemical alteration in patients with *ISCA2* gene mutations is hyperglycinemia. The main clinical features of patients affected by MMSD4 are spasticity (95%), optic atrophy (90%), nystagmus (65%), axial hypotonia (65%), and seizures (15%) [108]. The majority of the patients died during early childhood. Seven ISCA2 missense mutations (Table 1) have been so far reported, and they are mapped on a structural model of monomeric ISCA2 (Figure 7A).

The Gly77Ser mutation (c.G229 > A) was found in homozygosity in 20 cases from 18 different families [81,82,83]. In all patients after a normal initial development, a rapid deterioration between the ages of 2 and 7 months was observed, with neurodevelopmental regression, optic atrophy with nystagmus, and diffuse white matter disease [81,82,83]. A metabolic profile with elevated glycine, glutamate, lactate, and pyruvate levels in cerebral spinal fluid was reported for several patients [82]. Biochemical analysis, which was performed for only two (10%) patients, showed reduced complex I, II and IV activity [81,82]. The Gly77 residues is located in a highly conserved, functionally critical domain and is spatially close to in Fe-S cluster binding site (Figure 7A) [81]. Moreover, Gly77 possibly contributes to stabilize the complex between ISCA2 and IBA57. Gly77 is located in the loop containing the cluster ligand Cys79, is spatially close to one Arg residue involved in the electrostatic interactions discussed above in both A and B models (Figure 6), and is part of a triple GGG sequence highly conserved in ISCA2 eukaryotic homologues. These features suggest that the pathogenic mutation Gly77Ser might structurally affects the loop conformation, thus preventing or destabilizing Fe-S cluster binding both to ISCA2 and to the ISCA2-IBA57 complex as well as weakening the intermolecular electrostatic interaction, in this way disrupting cluster-mediated complex formation [81].

Leu52Phe was found in homozygosity in one patient, showing a mild phenotype, with isolated and non-progressive spastic paraplegia. The patient was still alive at the age of 12 [58]. Biochemical analysis showed that PDHc and α-KGDHc activities were only moderately decreased [58]. Leu52Phe mutation causes steric hindrance changes in a buried zone, likely destabilizing the tertiary structure. Arg105Gly was found in homozygosity in a patient that died before the age of 3 [58]. Biochemical analysis showed that the respiratory chain complex II assembly and activity were negatively affected by the missense mutation, with marked decreases in the SDHB subunit of complex II protein level. The enzymatic activities of PDHc and a-KGDHc were also decreased, as well as the IBA57 protein levels [58]. Arg105 is exposed to the surface on a β-sheet and the Arg105Gly mutation can severely destabilize the β-sheet and disrupt the electrostatic interaction with Glu100 present on the β-sheet plane (Figure 7B).

The Ala119Thr mutation (c.355G > A), affecting a highly conserved residue located in a highly conserved region of ISCA2, was found in homozygosity in a 7-months-old child of an Iranian consanguineous family [84]. The patient showed problems such as insomnia, irritability, muscle stiffness and hypotonia, but all general tests of the patient were normal. Metabolites analysis showed increased lactate levels and a relative increase in the choline with respect to N-acetyl aspartate in the patient’s brain, which is reported in several mitochondrial diseases [84]. The Ala119Thr mutation produces the change from hydrophobic to hydrophilic of a buried residue, thus likely negatively affecting the hydrophobic interactions and destabilizing the adjacent short α-helix.

Ala2Asp and Pro138Arg heterozygous mutations were found in a 11-year-old boy with a milder phenotype of MMSD4 [85]. The c.5C > A; p.Ala2Asp mutation was inherited from the mother and the c.413C > G; p.Pro138Arg from the father. The Ala2Asp mutation changes a highly conserved residue, located in the mitochondrial targeting peptide, while the Pro138Arg mutation affects the Fe-S cluster binding domain. Biochemical analysis revealed slightly elevated blood lactate and alanine levels and slightly elevated CSF lactate levels. Pro138 is located in a solvent exposed loop connecting the C-terminal β-hairpin, containing two Cys ligands, with the core domain of ISCA2. The Pro138Arg mutation, determining the addition of a charged residue, can have dramatic effects on the backbone conformation since proline determines a very well defined conformation and its substitution can have a major impact on the following β-hairpin that contains two out of the three Cys cluster binding ligands (Figure 7A).

The Ser112Gly missense mutation (c.334A > G) was found in combined heterozygosity with the frameshift mutation c.295delT/p.Phe99Leufs * 18 in a patient exhibiting diffuse hypotonia and a very rapid, fatal clinical course, who died at the age of 3 months [86]. High levels of lactate were found in CSF, associated to defects into complexes II and IV [86]. Although complex IV does not bind an iron–sulfur cluster, defects on it are common secondary effects of an impairment of the iron–sulfur protein biogenesis [109]. The Ser112Gly mutation introduces a loss of polarity on the α-helix where Ser112 is located, thus possibly destabilizing the α-helix by disrupting hydrogen bond interaction with Asp 109.

#### 3.2.5. Structural Aspects of Pathogenic Missense Mutations of ISCA1 Involved in MMDS5

Bi-allelic variants of *ISCA1* gene cause MMDS type 5 (MMSD5, MIM# 617613). Patients with MMSD5 have no psychomotor development and early onset seizures, with neurologic decline and spasticity [87]. Death usually occurs in early childhood.

**Figure 8:**
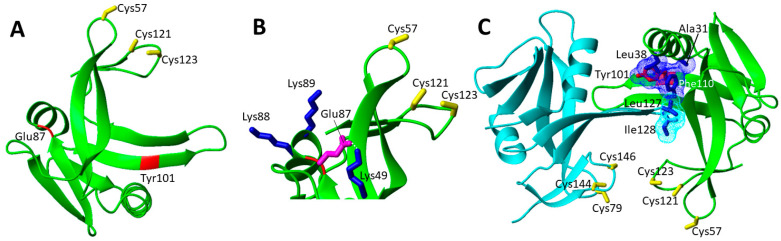
Pathogenic missense mutations mapped on the structural models of human ISCA1 and ISCA1-ISCA2 complex. (**A**) The backbone of the residues involved in pathogenic missense mutations of ISCA1 is colored in red on the structural model of monomeric apo ISCA1 obtained by AlphaFold2, and the sidechains of the three conserved cluster coordinating Cys residues are shown in yellow. (**B**) The hydrogen bond formed between the side-chains of Glu87 (in magenta) and Lys49 (in blue) is shown as a white dotted line. The sidechains of the positively charged Lys88 and Lys89 residues spatially close to Glu87 are also shown. (**C**) The sidechain of Tyr101 is shown in red on the hetero-dimeric apo ISCA1 (green)-ISCA2 (cyan) complex. The interacting hydrophobic patches formed by the sidechains of Tyr101 (in red), Phe110, Leu38 and Ala31 (all in blue) on ISCA1 protomer and by the sidechains of Leu127 and Ile138 (both in blue) on ISCA2 protomer are shown as Van der Waals surfaces in blue and cyan, respectively.

Only three missense homozygous variants of ISCA1have been so far reported (Table 1) and they are mapped on a structural model of monomeric ISCA1 (Figure 8A). The most common is the Glu87Lys mutation, which was found in homozygosis in five patients [87,88]. The missense substitution occurs at a highly conserved residue. Bioinformatic analyses of protein stability predict that this mutation destabilizes the ISCA1 protein [87]. Accordingly, this mutation determines a charge reversal, from negative to positive introducing a further positive charge in a region where other positive charges are present (Lys49, Lys88 and Lys 89, Figure 8B). The structural model of monomeric ISCA1 shows indeed that the mutation causes the disruption of a strong hydrogen bond between Glu87 and Lys49 (Figure 8B).

The Val10Gly pathogenic mutation was found in homozygosis, resulting in early onset leukodystrophy, severe spastic ataxia, lactic acidosis and respiratory defects [89]. The metabolic analysis showed high blood lactate and urine glycine levels. The mitochondrial proteins affected by this mutation were the respiratory chain complexes I and II, and LIAS. Indeed, the lipoylation of mitochondrial proteins was abolished in the patient carrying this mutation [89]. In contrast, respiratory chain complex III (binding a [2Fe-2S] cluster) was unaffected [89]. Val10 is located in the N-terminal region of ISCA1 corresponding to the uncleaved mitochondrial targeting sequence. For this reason, it is not possible to characterize it from the structural point of view since the prediction score of this region has a low reliability. However, consistently with the mutation location, experimental evidences showed that the Val10Gly mutation was found to affect the import, the stability and the function of ISCA1 [89], thus globally affecting the mitochondrial [4Fe-4S] proteins maturation.

The Tyr101Cys pathogenic mutation was found in homozygosis in one patient, for which psychomotor regression with loss of gait and language skills was reported, together with a tetrapyramidal spastic syndrome. Biochemical analysis of patient fibroblasts showed impaired lipoic acid synthesis and decreased activities of respiratory chain complexes I and II [90]. Expression and purification of the human ISCA1 showed a decreased stability of the [2Fe-2S] cluster in the mutated protein [90]. However, Tyr101 is far from the cluster binding site, and thus it should not directly affect the cluster binding ability of ISCA1 (Figure 8A). On the other hand, since Tyr101 is quite solvent exposed, it might play a role in the formation of both (i) the homo-dimeric ISCA1 species, which is able to bind the [2Fe-2S] cluster, and (ii) the complex that ISCA1 forms with ISCA2, which is responsible for the assembly of a [4Fe-4S] cluster. The analysis of a structural model of this hetero-complex [29] showed that the Tyr101Cys mutation might affect the ISCA1-ISCA2 complex formation, as Tyr101 is located in the extended β-strand involved in the protein complex interaction. Specifically, Tyr101 forms a hydrophobic patch together with Ala31, Phe110 and Leu 38 of ISCA1, which interacts with Leu 127 and Ile 128 of ISCA2 (Figure 8C). This mutation can thus significantly perturb such intermolecular contacts, weakening the formation of the hetero-complex and making it less abundant at the cellular level or less efficient in assembling the [4Fe-4S] clusters, whose level would be then lower. This picture fits well with the observed functionally impaired mitochondrial [4Fe-4S] proteins assembly. The proposed effect of this pathogenic mutation on destabilizing the ISCA1-ISCA2 complex, but without fully abolishing its function, agrees with the fact that the patient carrying this pathogenic mutation is 6 years old and the overall health status is improving [90].

#### 3.2.6. Structural Aspects of Pathogenic Missense Mutations of IND1 Involved in Mitochondrial Complex I Deficiency

Five missense (Table 1) and four non-missense disease-causing variants in IND1 (also named NUBPL) have been identified to date in patients affected by complex I deficiency. The amount of IND1 protein and of fully assembled complex I was strongly reduced in patients’ fibroblasts [91,94]. Clinical features include onset of neurological symptoms at the age of 3–18 months, global developmental delay, cerebellar dysfunction (including ataxia, dysarthria, nystagmus and tremor) and spasticity. Plasma and CSF lactate levels were high in most patients.

The Gly56Arg variant was found in heterozygosity with a complex gene rearrangement and a third paternal mutation, which ablated a consensus branch sequence (c.815-27T > C). Fibroblasts from the patient carrying these mutations exhibit a strong complex I defect, with low residual activity. However, the Gly56Arg variant may be unrelated to the disease occurrence, which is more likely caused by the other two non-sense mutations carried by the patient [92]. Indeed, the mutant protein was shown to be stable, does not have impaired mitochondrial import or cleavage, and can restore complex I activity in patient fibroblasts when overexpressed [93]. Even the available structural model of IND1 does not help to assess the pathogenicity of this mutation. Indeed, the mutation is located in a region with a low prediction score, thus with a low reliability.

**Figure 9:**
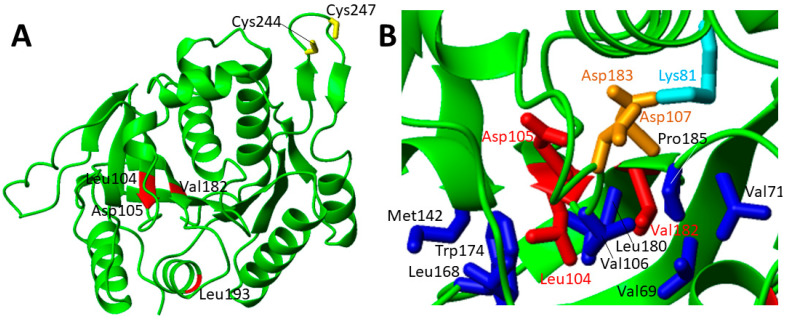
Pathogenic missense mutations mapped on the structural model of human IND1 obtained by AlphaFold2. (**A**) The backbone of the residues involved in pathogenic missense mutations of IND1 are colored in red on the ribbon structure of IND1, and the sidechains of the two conserved cluster coordinating Cys residues are shown in yellow. (**B**) The sidechains of Asp105, Leu104 and Val182 are shown in red. The Asp and Lys residues involved in the electrostatic interactions with Asp105 are shown in orange and cyan, respectively. The sidechains of the hydrophobic residues close in space to Leu104 and Val182 are shown in blue.

The other four pathogenic missense mutations are far from the twin-cysteine-residue motif that is required for the function of IND1 (Figure 9A). Thus, they are most likely involved in the destabilization of the protein fold.

The patients with the Asp105Tyr mutation in heterozygosity with Gly56Arg and with a third paternal mutation, which ablated a consensus branch sequence (c.815-27T > C), showed regression of speech and mobility with partial recovery, and motor problems due to ataxia. Plasma and cerebral spinal fluid lactate were elevated in patients carrying this IND1 variant [91,92,93]. Asp105 points to the interior of the protein and forms with Asp107 and Asp183 a peculiar buried negative patch that is balanced by the positive charge of the ε-amino group of Lys81 sidechain (Figure 9B). The carboxylate group of Asp105 is also engaged in one strong hydrogen bond with the backbone nitrogen of Asp107 that might contribute to stabilize the end of the extended parallel β-sheet. Thus, the Asp105Tyr mutation not only disrupts these interactions, but it also introduces a bulkier side chain that is very difficult to host in such a crowded environment. Overall, these effects are expected to destabilize the structure of the β-sheet. Two other pathogenic mutations, i.e., Leu104Pro and Val182Ala, are located on two adjacent parallel β-strands on the opposite side of the β-sheet where Asp105 is located (Figure 9B). p.Leu104Pro variant was found in trans with the c.815-27T > C branch-site mutation in three patients [94]. Clinical features include onset of neurological symptoms at the age of 3–18 months, general developmental delay, spasticity, ataxia, nystagmus and tremor. Brain MRI showed cerebellar atrophy. Mitochondrial function studies on patient fibroblasts showed significantly reduced spare respiratory capacity. Biochemical studies in a yeast model showed reduced complex I function [94]. p.Val182Ala variant was found in trans with the c.815-27T > C branch-site mutation in one patient [94]. The clinical features include spasticity, ataxia, nystagmus and tremor. No reduction of the complex I function are reported for this mutation [94]. Both Leu104Pro and Val182Ala mutations perturb hydrophobic interactions involving these two residues on one face of an extended β-sheet (Figure 9B). Furthermore, Leu104Pro mutation structurally perturbs two facing β-strands as a consequence of the proline substitution.

The p.Leu193Phe missense mutation was found in heterozygosity with the p.Gly56Arg missense mutation and with a c.815-27T > C branch-site mutation [91]. Early MRI of the patient showed prominent signal abnormalities and swelling of the corpus callosum [91]. Clinically, the patient exhibited insufficient gain or loss of motor skills and signs of cerebellar dysfunction before the second year of life [91]. On follow-up, signs of continuous regression were observed for the majority of patients [91]. Plasma and CSF lactate levels were elevated [91]. No biochemical analysis was reported for the patient. This mutation is located on the surface and, from the structural model of IND1, it does not appear so critical since the hydrophobic nature of the residue is maintained by the mutation.

#### 3.2.7. Structural Aspects of Pathogenic Missense Mutations of FDX2 Involved in Mitochondrial Muscle Myopathy

Pathogenic mutations of the *FDX2* gene cause episodic mitochondrial myopathy with or without optic atrophy and reversible leukoencephalopathy (MEOAL, OMIM number: 251900). MEOAL is an autosomal recessive neuromuscular disorder characterized by childhood onset of progressive muscle weakness and exercise intolerance [95]. The first case of mitochondrial myopathy associated to FDX2 was described in 2014 in a 15-year-old patient with recurrent myoglobinuria, lactic acidosis and slowly progressive muscle weakness due to a homozygous mutation affecting the start codon of FDX2 (c.1A > T, p.Met1Leu) and resulting in a severe reduction of the FDX2 protein levels [95]. The same mutation was found in 2017 in a patient with a similar muscular phenotype [58]. In these patients with this homozygous mutation in the *FDX2* gene, the enzymatic activity of the five respiratory chain complexes showed severely impaired functions in the Fe-S-dependent complexes I, II and III and mitochondrial aconitase [95]. Moreover, PDHc activity in muscle homogenate was decreased, whereas lipoamide dehydrogenase (subunit E3 of PDHc) activity was within the normal range.

The c.1A > T mutation causes the loss of the primary start codon ATG for Met1, which is replaced by a then un-translated triplet TTG coding for Leu. When a mutation alters the start codon, the scanning mechanism should activate initiation from the next downstream ATG [110]. The next putative in frame start codon is present only 14 codons downstream coding for Met5; however, this methionine residue completely lacks the surrounding Kozak sequence, making it very unlikely alternative initiation site. In agreement with the predicted deleterious effect of the mutation on the translation of the protein, immunoblot assay of the patients’ muscle and fibroblasts demonstrated lack of FDX2 band as compared with controls [95].

In 2018, another homozygous missense mutation in FDX2 (c.431C > T, p.Pro144Leu) was described in six patients from two unrelated families with autosomal recessive inheritance of a complex neurological phenotype involving early onset optic atrophy followed in the first or second decade of life by progressive myopathy, recurrent episodes of cramps, myalgia, muscle weakness and axonal polyneuropathy [96]. While no difference in FDX2 mRNA expression between patients and controls was observed using muscle samples of the patients with homozygous c.431C > T mutation, a severe reduction of FDX2 protein levels was observed in the same patients as compared to controls.

The available crystal structure of [2Fe-2S] FDX2 (PDB ID 2Y5C) showed that Pro144 is located in a solvent-exposed loop being only capped by the C-terminus. This loop connects an α-helix with a β-strand and is about 10 Å far from the [2Fe-2S] cluster. The pathogenic mutation Pro144Leu, although remaining hydrophobic, introduces a bulkier side chain that might cause steric hindrance on the residues of the spatially close C-terminus, in such a way to possibly displace it towards the cluster binding site.

## 4. Conclusions

Mutations in genes involved in the mitochondrial ISC assembly machinery cause severe disorders in humans. In particular, mutations in any of the genes involved in the assembly and transfer of mitochondrial [4Fe-4S] clusters are found to have a relevant impact in iron–sulfur protein assembly diseases. Many of them are fatal, sometimes already in early childhood. These evidences raise the question of how pathogenic mutations that affect a specific and unique pathway can impact human health so dramatically. This review thus aimed at providing an overview on how missense mutations on these genes can generate a quite wide spectrum of human diseases that are, however, all associated with the molecular misfunction of accessory proteins. The latter are indeed in charge of a specific biosynthetic pathway responsible for the biogenesis of [4Fe-4S] mitochondrial proteins. Our molecular analysis showed that pathogenic point mutations can affect the function of the protein in different ways, by impairing protein folding or cluster binding as well as by negatively affecting protein–protein recognition or preventing complex formation. These molecular effects can be active in parallel in such a way to produce a strong decrement in the efficiency of generating the required cellular levels of functionally active mitochondrial [4Fe-4S] target proteins.

## Figures and Tables

**Table 1:** Missense mutations of the late-acting ISC components with the associated mitochondrial disorders.

Gene/ Protein	Missense Mutation	Predicted Protein Mutations	Associated Disease	References
GLRX5	c.301A > C; c.443T > C	p.Lys101Gln; p.Leu148Ser	Nonsindromic sideroblastic anemia 3	[51]
	c.200G > A; c.383T > A	p.Cys67Tyr; p.Met128Lys	Nonsindromic sideroblastic anemia 3	[52]
NFU1	c.545G > A; c.545G > A	p.Arg182Gln; p.Arg182Gln	MMDS1	[43]
	c.622G > T; c.622G > T	p.Gly208Cys; p.Gly208Cys	MMDS1	[55,56]
	c.565G > A; c.565G > A	p.Gly189Arg; p.Gly189Arg	MMDS1	[57]
	c.179G > T; c.179G > T	p.Phe60Cys; p.Phe60Cys	MMDS1	[58]
	c.545G > A; c.622G > T	p.Arg182Gln; p.Gly208Cys	MMDS1	[59]
	c.544C > T; c.622G > T	p.Arg182Trp; p.Gly208Cys	MMDS1	[60]
	c.629G > T; c.622G > T	p.Cys210Phe; p.Gly208Cys	MMDS1	[58]
	c.565G > A; c.622G> T	p.Gly189Arg; p.Gly208Cys	MMDS1	[58,61]
	c.565G > A; c.629G > T	p.Gly189Arg; p.Cys210Phe	MMDS1	[62]
	c.565G > A; c.568G > A	p.Gly189Arg; p.Gly190Arg	MMDS1	[63]
	c.62G > C; c.622G > T	p.Arg21Pro; p.Gly208Cys	MMDS1	[63]
	c.299C > G; c.398T > C	p.Ala100Gly; p.Leu133Pro	MMDS1	[64]
	c.721G > T; c.303_369del	p.Val241Phe; ?	MMDS1	[65]
BOLA3	c.200T > A; c.200T > A	p.Ile67Asn; p.Ile67Asn	MMDS2	[66,67]
	c.287A > G; c.287A > G	p.His96Arg; p.His96Arg	MMDS2	[68,69,70]
	c.295C > T; c.295C > T	p.Arg99Trp; p.Arg99Trp	MMDS2	[71]
	c.176G > A; c.136C > T	p.Cys59Tyr; p.Arg46 *^a^	MMDS2	[72]
IBA57	c.706C > T; c.706C > T	p.Pro236Ser; p.Pro236Ser	MMDS3	[73]
	c.941A > C; c.941A > C	p.Gln314Pro; p.Gln314Pro	MMDS3	[74]
	c.286T > C; c.188G > A	p.Tyr96His; p.Gly63Asp	MMDS3	[75,76]
	c.316A > G; c.286T > C	p.Thr106Ala; p.Tyr96His	MMDS3	[75,76]
	c.738C > G; c.316A > G	p.Asn246Lys; p.Thr106Ala	MMDS3	[58]
	c.757G > C; c.316A > G	p.Val253Leu; p.Thr106Ala	MMDS3	[58]
	c.335T > G; p.437G > C	p.Leu112Trp; p.Arg146Pro	MMDS3	[58]
	c.335T > C; c.588dup	p.Leu112Ser; p.Arg197Alafs	MMDS3	[77]
	c.386A > T; c.731A > C	p.Asp129Val; p.Glu244Ala	MMDS3	[78]
	c.436C > T; c.436C > T	p.Arg146Trp; p.Arg146Trp	MMDS3	[79]
	c.586T > G;c.686C > T	p.Trp196Gly; p.Pro229Leu	MMDS3	[73]
	c.656 > G; c.706C > T	p.Tyr219Cys; p.Pro236Ser	MMDS3	[71]
	c.701A > G; c.782T > C	p.Asp234Gly; p.Ile261Thr	MMDS3	[75,76]
	c.738C > G; c.802C > T	p.Asn246Lys; p.Arg268Cys	MMDS3	[71]
	c.286T > C; c.754G > T	p.Tyr96His; p.Gly252Cys	MMDS3	[75,76]
	c.323A > C; c.150C > A	p.Tyr108Ser; pCys50 *^a^	MMDS3	[78]
	c.87insGCCCAAGGTGC; c.313C > T	p.Arg30Alafs; p.Arg105Trp	MMDS3	[73]
	c.236C > T; c.307C > T	p.Pro79Leu; p.Gln103 *^a^	MMDS3	[76]
	c.580A > G; c.286T > C	p.Met194Val; p.Tyr96His	MMDS3	[80]
ISCA2	c.154C > T; c.154C > T	p.Leu52Phe; p.Leu52Phe	MMDS4	[58]
	c.313A > G; c.313A > G	p.Arg105Gly; p.Arg105Gly	MMDS4	[58]
	c.G229 > A; c.G229 > A	p.Gly77Ser; p.Gly77Ser	MMDS4	[81,82,83]
	c.355G > A; c.355G > A	p.Ala119Thr; p.Ala119Thr	MMDS4	[84]
	c.5C > A; c.413C > G	p.Ala2Asp; p.Pro138Arg	MMDS4	[85]
	c.295delT; c.334A > G	p.Phe99Leufs*18; ^b^ p.Ser112Gly		[86]
ISCA1	c.259G > A; c.259G > A	p.Glu87Lys; p.Glu87Lys	MMDS5	[87,88]
	c.29T > G; c.29T > G	p.Val10Gly; p.Val10Gly	MMDS5	[89]
	c.302A > G; c.302A > G	p.Tyr101Cys; p.Tyr101Cys	MMDS5	[90]
IND1	c.815-27T > C; c.G166 > A	p.Asp273Glnfs*31; ^b^ p.Gly56Arg	Complex I deficiency	[91,92,93]
	c.313G > T; c.166G > A; c.815-27T > C	p.Asp105Tyr; p.Gly56Arg; p.Asp273Glnfs*31 ^b^	Complex I deficiency	[91,92,93]
	c.579A > C; c.G166 > A	p.Leu193Phe; p.Gly56Arg	Complex I deficiency	[91,92,93]
	c.311T > C; c.815-27T > C	p.Leu104Pro; p.Asp273Glnfs*31 ^b^	Complex I deficiency	[94]
	c.815-27T > C; c.545T > C	p.Val182Ala; p.Val182Ala	Complex I deficiency	[94]
FDX2	c.1A > T; c.1A > T	p.Met1Leu; p.Met1Leu	MEOAL	[95]
	c.431C > T; c.431C > T	p.Pro144Leu; p.Pro144Leu	MEOAL	[96]

^a^ The “*” symbol indicates a nonsense mutation; ^b^ frameshift mutation
